# Effect of the NH_3_ Precursor on the Properties and Temperature-Pressure Response Mechanisms of Low-Temperature PECVD Silicon Nitride Film

**DOI:** 10.3390/ma19132905

**Published:** 2026-07-06

**Authors:** Zhen Tang, Peng Yu, Yanli Qi, Zhuo Wang, Jianping Ning, Zhaohui Ren

**Affiliations:** 1 School of Mechanical Engineering and Automation, Northeastern University, Shenyang 110819, China; 2 Piotech, Inc., 900 Shuijia Street, Shenyang 110000, China; peng.yu@sy.piotech.cn (P.Y.); yanli.qi@sh.piotech.cn (Y.Q.); zhuo.wang@sh.piotech.cn (Z.W.); jianping.ning@sh.piotech.cn (J.N.)

**Keywords:** low-temperature PECVD, silicon nitride film, chamber pressure, temperature, NH_3_ precursor

## Abstract

**Highlights:**

- Compared to using pure SiH_4_/N_2_ system, the lower dissociation energy of NH_3_ doubles the deposition rate and improves uniformity.
- Pressure-dependent NH_3_ dissociation modulates stress transition from compressive to tensile.
- Process pressure dominates film chemical bonding evolution over substrate temperature.

**Abstract:**

The integration of advanced semiconductor architectures strictly mandates process thermal budgets below 200 °C, positioning low-temperature PECVD of silicon nitride (SiN_x_) film as a critical layer. However, SiN_x_ film deposited at sub-200 °C inherently exhibits sluggish deposition kinetics and degraded spatial uniformity. To overcome these bottlenecks, this study systematically investigates the regulatory mechanisms of the NH_3_ precursor within SiH_4_/N_2_-based plasmas under varying chamber pressures and substrate temperatures. The results show that the introduction of NH_3_ at 2.1 Torr, leveraging its facile plasma dissociation, drastically enhances the deposition rate from 18.2 to 39.1 Å/s and improves thickness uniformity by 1.07%. Meanwhile, NH_3_ supplies abundant highly reactive radicals that elevate the refractive index and reinforce compressive stress. Furthermore, film properties exhibit a higher sensitivity to pressure than to temperature, primarily due to the pronounced influence of pressure on plasma dynamics and collision frequencies, whereas the effect of temperature remains comparatively minor. This phenomenon is clearly demonstrated by the Si–H and N–H content. This study validates that operating at low chamber pressures maximizes the collision-free travel distance of SiN_x_ radicals, providing an optimized and quantified process window for high-volume manufacturing of low-temperature SiN_x_ film.

## 1. Introduction

With the continuous evolution of integrated circuit (IC) architectures and the rapid development of advanced packaging paradigms, the semiconductor industry faces a surging demand for high-performance SiN_x_ film. SiN_x_ films have emerged as a critical component due to their outstanding dielectric characteristics and remarkable chemical inertness [1,2]. Traditionally, the growth of these film relies on high-temperature thermal chemical vapor deposition (CVD) or conventional plasma-enhanced chemical vapor deposition (PECVD) at approximately 400 °C. However, emerging frontiers—such as back-end-of-line (BEOL) interconnects, 3D heterogeneous integration, flexible electronics, and power device packaging—are highly sensitive to thermal budgets [3]. These applications strictly mandate that processing temperatures be suppressed to below 200 °C. Consequently, exploring low-temperature PECVD techniques for precise SiN_x_ deposition has become an essential technological hurdle [4,5].

In typical PECVD processes, SiN_x_ layers are grown using precursor gas mixtures, predominantly SiH_4_/NH_3_/N_2_ or SiH_4_/N_2_ [6,7]. Once energized by radio-frequency (RF) plasma, these precursor molecules undergo ionization and dissociate into highly reactive intermediate fragments (e.g., SiH_x_ and NH_x_ radicals) [8,9]. These active species are then transported to the substrate, where they adsorb, migrate, and polymerize to construct a stable Si–N cross-linked network [10]. Nevertheless, dropping the process temperature from the conventional 400 °C to the sub-200 °C regime introduces two major obstacles. First, the lack of sufficient thermal activation energy severely hinders both precursor fragmentation and surface chemical reactions, leading to a drastically lowered deposition rate. This bottleneck directly impacts wafer throughput and manufacturing cost-efficiency [11]. Second, the restricted thermal mobility of adsorbed atoms on the surface causes severe degradation in film uniformity, resulting in undesirable fluctuations in thickness, chemical composition, and internal stress [12,13]. Such spatial variations ultimately compromise the structural integrity and electrical yield of the fabricated devices. Hence, overcoming the twin hurdles of sluggish deposition kinetics and inadequate uniformity is indispensable for scaling low-temperature PECVD up to high-volume manufacturing.

Previous investigations into 400 °C PECVD processes have documented that adjusting variables like chamber pressure, RF power, and gas flow ratios can effectively tailor film characteristics [14]. For example, the improved film uniformity, characterized by a 50% reduction in non-uniformity, was achieved by reducing the process pressure by 0.2 Torr [15]. This enhancement is attributed to the increased mean free path of gas molecules, which promotes more uniform precursor diffusion across the substrate. Concurrently, the deposition rate was significantly enhanced by approximately 65% by increasing the NH_3_ flow rate from 25 to 100 sccm, owing to the heightened concentration of reactive nitrogen species [16]. These findings suggest that process optimization can theoretically reconcile the trade-off between growth rate and uniformity. However, a comprehensive understanding of the reaction mechanisms, specifically in the sub-200 °C window, remains largely absent. The current literature offers fragmented insights, mostly derived from idealized lab-scale models that deviate significantly from realistic industrial process windows [13]. Conversely, industrial implementations often bypass rigorous investigations into underlying physicochemical kinetics. It is noteworthy that the mechanistic influence of the same parameter on film stress may differ across process windows. For example, Li’s team found that increasing chamber pressure leads to a shift in film stress from −200 MPa to 200 MPa [17]. In contrast, our team’s single-variable experiments within a producible process window revealed a gradual decline in tensile stress with rising pressure [15]. This phenomenon may arise from multi-physical field coupling effects during deposition. Consequently, process development necessitates systematic analysis of key parameters’ impacts on film stress and other properties for specific process windows [18,19]. Therefore, bridging this gap by conducting mechanistic studies under practical mass-production conditions is of paramount importance.

In this work, we systematically explored the low-temperature PECVD growth of SiN_x_ film utilizing SiH_4_/NH_3_/N_2_ systems (with and without NH_3_). The impacts of chamber pressure and substrate temperature on crucial film characteristics, including deposition rate (*DR*), thickness non-uniformity (*NU*), refractive index (*RI*), and intrinsic stress, were thoroughly evaluated. Furthermore, the microstructural evolution of chemical bonds was analyzed to quantitatively assess the functional role of the NH_3_ precursor, elucidating the distinct kinetic behaviors between the two gas systems. This research offers a viable engineering pathway for optimizing the yield and quality of low-temperature SiN_x_ film while providing a deeper theoretical perspective on sub-200 °C plasma deposition dynamics.

## 2. Materials and Methodology

### 2.1. Preparation of Silicon Nitride Film

The SiN_x_ film was synthesized utilizing an industrial-scale PECVD platform. Ultra-high-purity-grade gases (>99.99%), including N_2_, SiH_4_, and NH_3_, were employed as reactants. A silicon nitride film with a thickness of approximately 2000 Å was deposited on a silicon wafer having a diameter of 300 mm. Before deposition, the wafer was preheated to ensure process stability. Guided by prior optimization trials from our research group, the standard process parameters were established and are summarized in Table 1. Two process gas chemistries were employed: (1) the SiH_4_/N_2_ system, with a SiH_4_ flow rate of 300 sccm and a N_2_ flow rate of 10,000 sccm; and (2) the SiH_4_/N_2_/NH_3_ system, wherein an additional NH_3_ flow of 200 sccm was introduced while maintaining the SiH_4_ and N_2_ flow rates unchanged. All gases were uniformly injected into the reaction chamber through the upper electrode. The parallel-plate electrode gap was set to 16 mm, and the process pressure was maintained at 3 Torr. The selected temperature and the pressure values were determined based on the established practical process window for this deposition technique. Pressure is regulated by adjusting the valve aperture, while the electrode gap is defined as the distance between the upper and lower electrode plates.

To systematically decouple the effects of operational variables, two independent series of experiments were executed: one varying the substrate temperature (140, 150, 160, 170, and 180 °C) and another modifying the chamber pressure (2.1, 2.4, 2.7, 3.0, 3.3, 3.6, and 3.9 Torr). The range of temperature and pressure conditions explored was designed to utilize the available tuning space of the production equipment, allowing for systematic optimization while adhering to operational constraints.

### 2.2. Characterization Methods

Deposition rate was used to evaluate the growth kinetics of the film. An Aleris 8500 optical metrology tool (KLA Tencor, Milpitas, CA, USA) was utilized to measure film thickness. For statistical reliability, six individual wafers were scanned for each experimental condition. The *DR* was derived using Equation (1), where *THK* stands for the average thickness across the six samples, and *t* is the effective processing time.

(1) DR(Å/s)=THK/t

To evaluate the quality of the deposited film, the non-uniformity of the film was tested. The spatial variation in film thickness was mapped utilizing the same Aleris 8500 system based on a standard 49-point measurement protocol. The area within a 3 mm margin was not tested, and 49 points were evenly distributed across the wafer. The non-uniformity percentage was computed via Equation (2), where *σ_THK_* and *μ_THK_* denote the standard deviation and the arithmetic mean of the measured points, respectively.

(2) $$NU(\%)=\frac{\sigma_{THK}}{\mu_{THK}}\times 100\%$$

The refractive index is used to characterize the optical properties of the film, which was assessed by (KLA Tencor, Milpitas, CA, USA). The refractive index of the film was determined at a wavelength of 633 nm. The reported values represent the statistical mean derived from testing six independent wafers per process condition.

The residual mechanical stress within the deposited film was analyzed employing a T910 system (Skyverse, Shenzhen, China). The scanning mode is line scanning. Six wafers were evaluated per batch. The intrinsic stress was deduced from the variation in wafer curvature before and after the PECVD process, strictly adhering to the Stoney Equation.

**Figure 1:**
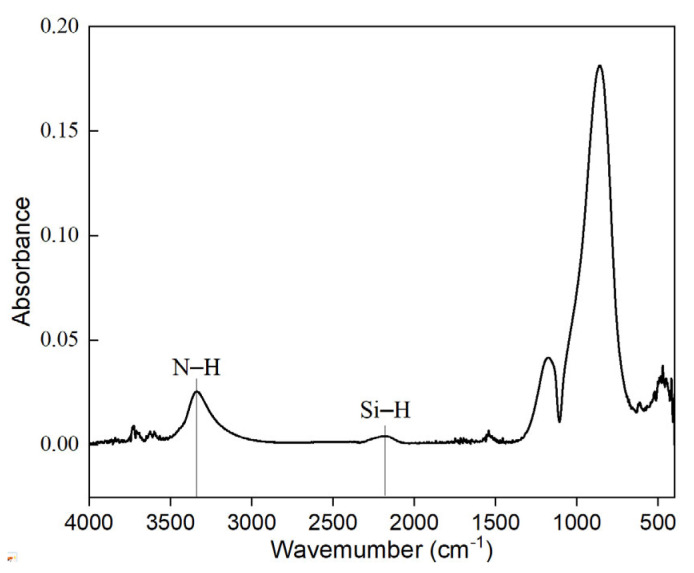
Schematic diagram of the FT-IR spectroscopy of silicon nitride film.

The internal bonding structures of the SiN_x_ matrices were probed via Nicolet™ iS50 Fourier-transform infrared (FT-IR) spectroscopy (Waltham, MA, USA), spanning a wavenumber spectrum from 400 to 4000 cm^−1^. The number of scans was 32, and the measurement type was attenuated total reflectance. The Lanford–Rand method [20], as recommended by Thermo Scientific (Waltham, MA, USA)., was applied based on the Si–H and N–H peaks in Figure 1. The FT-IR spectroscopy of the SIH_4_/NH_3_/N_2_ system is shown in Figure 1, and the deposition parameters are as shown in Table 1. The film thickness measured was approximately 2000 Å. In addition, the thickness was considered when calculating the Si–H and N–H contents, thereby eliminating the influence of thickness variations. By integrating the areas under the respective absorption peaks, the absolute hydrogen content (*C_H_*) and the relative proportion of Si–H to N–H bonds (*R_H_*) were calculated using Equations (3) and (4). Here, *C_Si–H_* and *C_N–H_* correspond to the content of the Si–H and N–H vibrational modes.

(3) $$C_{H}=C_{Si-H}+C_{N-H}$$

(4) $$R_{H}=C_{Si-H}/C_{N-H}$$

## 3. Results and Discussion

### 3.1. Deposition Rate

**Figure 2:**
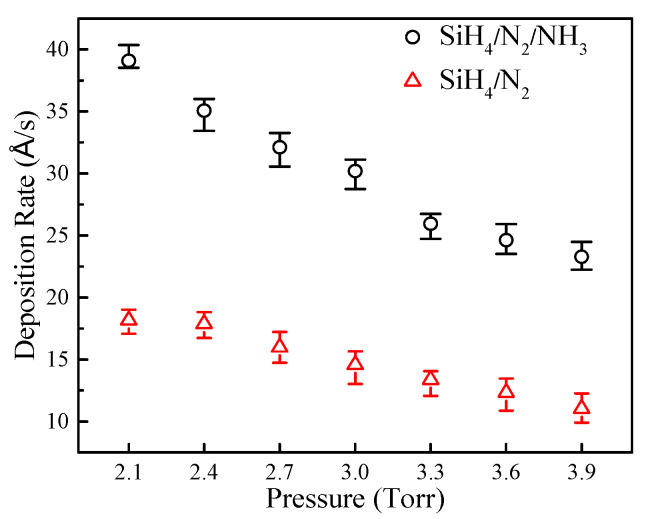
Variation in deposition rate with pressure for the two systems.

Figure 2 shows the dependence of the SiN_x_ film *DR* on chamber pressure for both the SiH_4_/N_2_ and SiH_4_/N_2_/NH_3_ systems. As depicted in Figure 1, a continuous decline in growth kinetics is observed for both systems as the chamber pressure scales up. Specifically, increasing the pressure from 2.1 Torr to 3.9 Torr drives the *DR* of the NH_3_-inclusive system down from 39.1 Å/s to 23.3 Å/s, while the NH_3_-free system drops from 18.2 Å/s to 11.0 Å/s. Despite the higher volumetric concentration of reactants at elevated pressures, the actual deposition speed is retarded. This counterintuitive trend originates from the intensified molecular collision frequency at higher pressures, which drastically truncates the mean free path of gas-phase species [21]. As pressure increases from 2.1 to 3.9 (an 86% increase), the mean free path decreases by approximately 46%. This reduction in mean free path corresponds to decreased molecular diffusivity, ultimately contributing to the observed decline in deposition rate. Meanwhile, the kinetic energy of the particles drops, surface diffusion is hindered, and the effective delivery of active radicals to the substrate is suppressed. Moreover, under identical pressure conditions, the addition of NH_3_ yields a substantially faster deposition rate compared to the pure N_2_ baseline. The reason is that the dissociation energy of NH_3_ (−341 kJ/mol) is lower than that of N_2_ (−945 kJ/mol) [22]. The deposition rate differential (Δ*DR*) shows that as pressure climbs to 3.9 Torr, Δ*DR* shrinks from 20.9 Å/s to 12.2 Å/s. This indicates that while NH_3_ inherently boosts film formation, this kinetic advantage is significantly neutralized by the restricted molecular mobility and shorter diffusion lengths prevalent in high-pressure environments [22].

**Figure 3:**
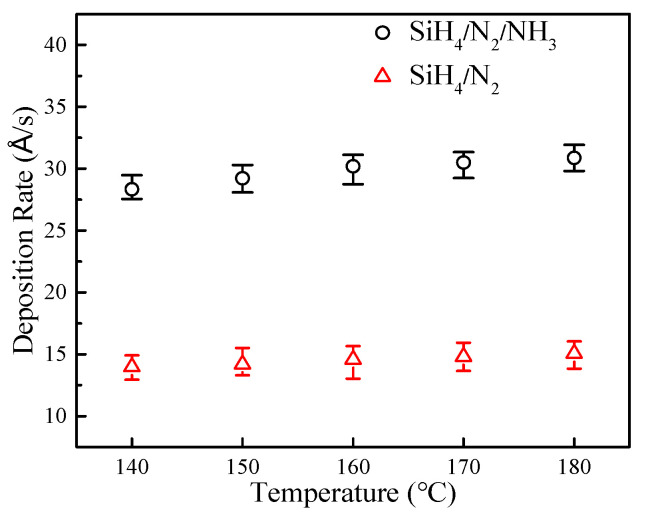
Variation in deposition rate with temperature for the two systems.

The impact of substrate temperature on the deposition speed for both gas configurations is captured in Figure 3. Within the investigated thermal window, the growth rates exhibit only a marginal upward trajectory. Elevating the temperature from 140°C to 180°C results in a minor rate increase from 28.3 Å/s to 30.9 Å/s for the SiH_4_/N_2_/NH_3_ system and from 14.0 Å/s to 15.1 Å/s for the SiH_4_/N_2_ system. This weak thermal dependence stems from the narrow temperature range employed and the fact that, at sub-200°C, film growth is overwhelmingly driven by plasma-induced fragmentation rather than thermal activation [23,24]. A gradual widening of Δ*DR* is observed with rising temperature. This suggests that moderate thermal energy supplements the surface mobility of adsorbed radicals and assists in the plasma-phase dissociation of NH_3_, yielding a more pronounced kinetic enhancement at 180°C.

In summary, while incorporating NH_3_ fundamentally accelerates SiN_x_ deposition, unlocking its full potential necessitates careful tuning of the thermodynamic and pressure parameters. The empirical evidence points to a combination of lower pressure and the upper end of the low-temperature regime as the optimal window. Lowering the pressure preserves the mean free path and reactant transport, whereas mild heating augments surface reactivity and NH_3_ fragmentation, thereby maximizing the deposition efficiency.

### 3.2. Non-Uniformity

**Figure 4:**
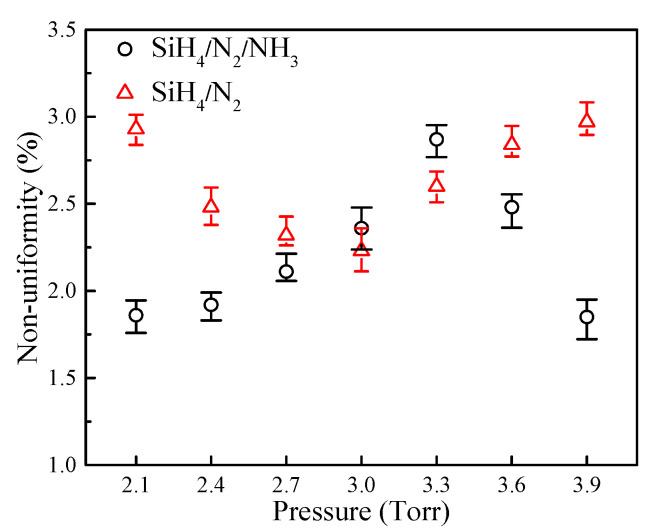
Variation in non-uniformity with pressure for the two systems.

Figure 4 maps the trajectory of thickness non-uniformity as chamber pressure shifts from 2.1 to 3.9 Torr. Interestingly, the two systems display opposing trends. For the SiH_4_/N_2_/NH_3_ system, *NU* initially deteriorates from 1.86% to 2.87% before recovering to 1.85%. Conversely, the SiH_4_/N_2_ system improves from 2.93% to 2.23%, only to worsen again to 2.97% at higher pressures. This difference may be determined by the subtle interaction between the plasma decomposition kinetics and the diffusion of free radicals. In the absence of NH_3_, moderate pressures achieve an optimal balance between mean free path and spatial reactant distribution, minimizing thickness variations. However, excessive pressure eventually stifles gas diffusion, deteriorating uniformity. When NH_3_ is present, intermediate pressures rapidly generate an abundance of highly reactive NH_x_ radicals. Their aggressive local consumption creates a center-to-edge “depletion effect,” spiking the non-uniformity. As pressure maxes out at 3.9 Torr, enhanced collisional quenching of excess radicals, combined with longer gas residence times, surprisingly fosters a more homogeneous reactant distribution, thereby rescuing the uniformity. Notably, at the minimal pressure of 2.1 Torr, the *NU* values of the SiH_4_/N_2_ and SiH_4_/N_2_/NH_3_ systems are 1.86% and 2.93%, respectively, with a difference of 1.07 percentage points. NH_3_ incorporation delivers the starkest improvement, which underscores that a low-pressure regime is critical for ensuring the uniform dispersion of highly reactive fragments.

**Figure 5:**
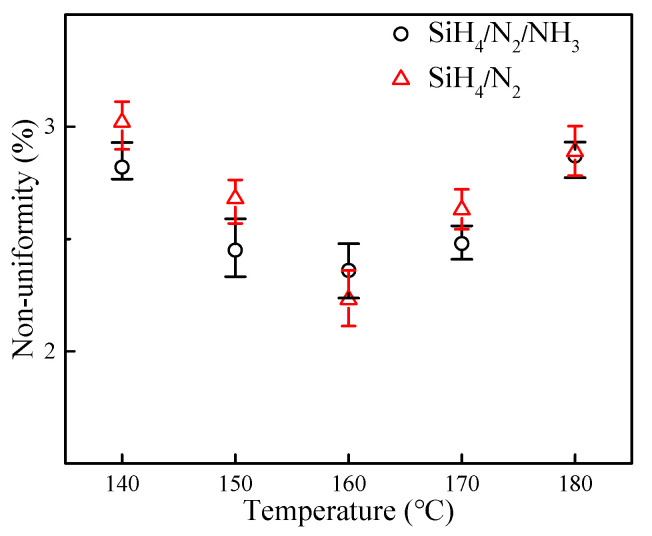
Variation in non-uniformity with temperature for the two systems.

Figure 5 examines the thermal response of film *NU*. In contrast to the pressure-induced divergence, varying the temperature from 140 °C to 180 °C forces both systems into an identical U-shaped profile. The *NU* of the SiH_4_/N_2_/NH_3_ system dips from 2.82% to 2.36% before climbing to 2.87%, mimicking the SiH_4_/N_2_ system’s drop from 3.0% to 2.23%, followed by a rise to 2.89%. This behavior is dictated by the rivalry between adatom surface migration and local cross-linking rates. At moderate thermal inputs, increased surface mobility allows atoms to fill micro-voids, enhancing macroscopic uniformity. However, as temperatures approach 180 °C, shifts in the sticking coefficients of certain radicals cause localized Si–N bonding to outpace surface diffusion. Furthermore, slight thermal gradients across the wafer can become magnified, inducing center-to-edge kinetic mismatches that degrade uniformity. The specific influence of NH_3_ on *NU* remains marginal across temperatures, which indicates that surface diffusion mechanisms in this cold-plasma regime are relatively insensitive to NH_3_ addition, being constrained by the low thermal budget.

Ultimately, the effect of NH_3_ on structural homogeneity is dominated by pressure-dependent mean-free-path dynamics rather than temperature-driven adatom migration. Operating at a low pressure of 2.1 Torr is identified as the prerequisite for suppressing the radical depletion effect, allowing the NH_3_ system to deliver both rapid growth and superior spatial uniformity.

### 3.3. Refractive Index

**Figure 6:**
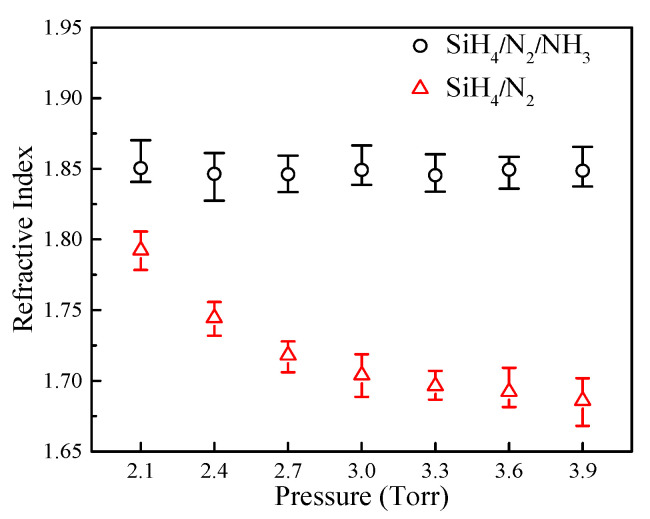
Variation in refractive index with pressure for the two systems.

The relationship between chamber pressure and the *RI* of the SiN_x_ film is charted in Figure 6. Without NH_3_, the *RI* experiences a steady decay from 1.792 at 2.1 Torr down to 1.686 at 3.9 Torr. This degradation implies that heightened molecular collisions at higher pressures dissipate the kinetic energy of bombarding ions. The weakened ion peening effect results in a porous, less dense matrix, thus lowering the optical density. Conversely, the introduction of NH_3_ not only elevates the overall *RI* but also renders it remarkably immune to pressure fluctuations [25]. The *RI* enhancement (Δ*RI*) provided by NH_3_ drastically expands from 0.058 to 0.163 as pressure increases. This stabilization arises from the facile decomposition of NH_3_ into abundant NH_x_ radicals and atomic hydrogen. These highly reactive species accelerate the formation of dense, high-quality Si–N networks. The vigorous chemical kinetics introduced by NH_3_ effectively supersede the physical densification typically achieved through ion bombardment, thereby counteracting the adverse effects of elevated pressure [26,27]. The increase in refractive index upon NH_3_ addition is attributed to the same mechanism. In the absence of NH_3_, N_2_ is not easily dissociated, resulting in numerous Si dangling bonds. Consequently, a porous film is formed, and its refractive index is decreased. With the introduction of NH_3_, which is more readily dissociated, the formation of Si–N bonds is promoted, leading to a denser network structure and an increase in the refractive index.

**Figure 7:**
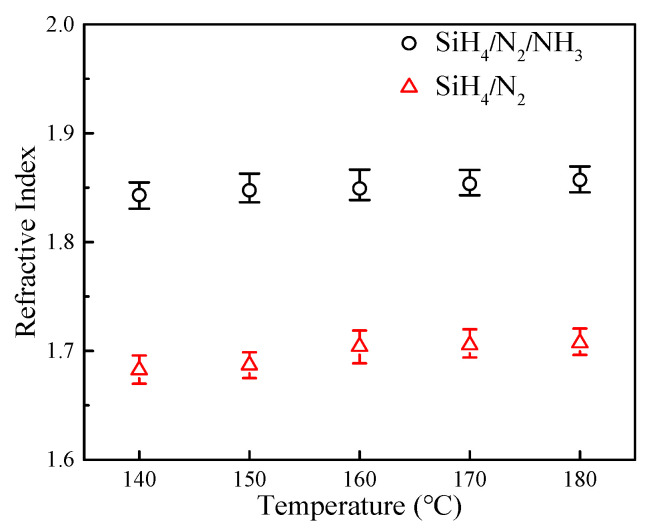
Variation in refractive index with temperature for the two systems.

Figure 7 details the thermal dependence of the refractive index. Across the 140 °C to 180 °C span, the *RI* for both systems remains virtually static. Macroscopically, the refractive index serves as an indicator of film stoichiometry (Si/N ratio) and network compaction. In the highly restricted thermal environment of this study, the supplied heat is grossly inadequate to trigger hydrogen desorption or facilitate extensive lattice reconstruction. Therefore, the internal chemical composition ratio and structural density are mainly influenced by the chemical reactions of the plasma phase. Substrate temperature, therefore, plays a negligible role in defining the optical properties of these low-temperature films.

In conclusion, NH_3_ acts as a powerful source of active radicals that heavily fortifies the refractive index. Because the film chemistry is dictated by these abundant reactive species, the optical density displays remarkable resilience against pressure variations and remains entirely decoupled from thermal fluctuations within the sub-200°C window.

### 3.4. Stress

**Figure 8:**
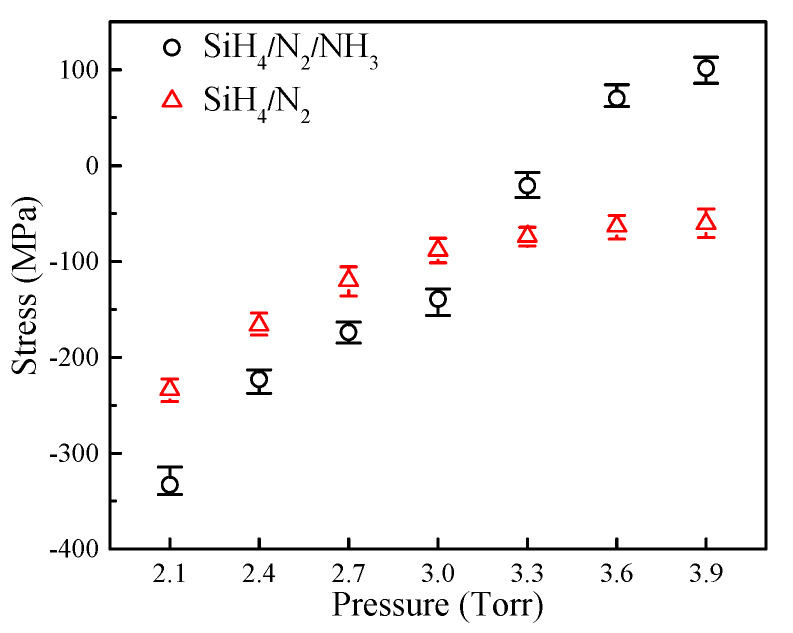
Variation in stress with pressure for the two systems.

Figure 8 charts the evolution of residual film stress in response to varying chamber pressures for the two systems. As the ambient pressure escalates from 2.1 Torr to 3.9 Torr, a definitive mechanical inversion from a compressive state (negative values) to a tensile state (positive values) manifests in both systems. Specifically, the internal stress of the SiH_4_/N_2_ system shifts dramatically from −233.8 MPa to 60.4 MPa, whereas the SiH_4_/N_2_/NH_3_ film undergoes a transition from −332.9 MPa to 101.6 MPa. This overall stress variation can be influenced by the ion sputtering mechanism within the plasma environment. In the low-pressure regime, the extended collision-free travel distance allows ions to maintain substantial kinetic energy, heavily bombarding the growing surface. This intense physical compaction densifies the structure, thereby embedding significant compressive strain. Conversely, as pressure increases, enhanced collision frequencies dissipate ion kinetic energy prior to substrate impact. The deprivation of this densifying bombardment yields a comparatively relaxed and porous micro-architecture, naturally favoring the accumulation of tensile stress [28,29,30].

At the lower pressure boundary, the inclusion of NH_3_ exacerbates the compressive stress. This occurs because the abundant reactive radicals supplied by NH_3_ facilitate the rapid construction of a highly cross-linked and compact Si–N network—a structural evolution that aligns perfectly with the previously observed surge in optical refractive index. Intriguingly, in the high-pressure domain, NH_3_ incorporation actively drives the film further into the tensile regime. This behavior can be rationalized by the rapid chemical kinetics occurring under low ion-energy conditions. Without enough bombardment to compact the film, reaction by-products (such as volatile H or bulky NH_x_ fragments) become sterically entrapped within the loosely packed network. The subsequent localized structural contraction and the generation of microscopic voids directly contribute to elevated tensile strain.

**Figure 9:**
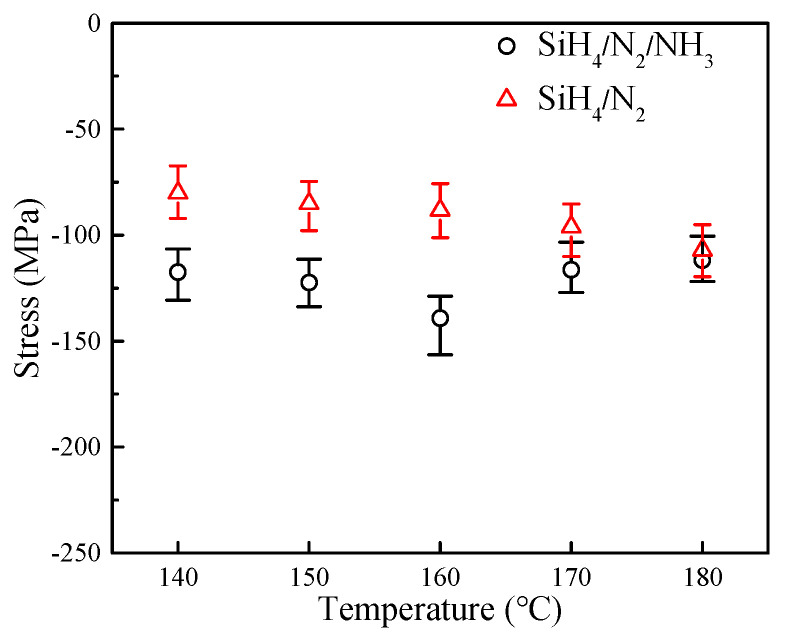
Variation in stress with temperature for the two systems.

The thermal dependence of internal stress is mapped in Figure 9. Adjusting the substrate temperature from 140 °C to 180 °C induces a gradual amplification of compressive stress in the SiH_4_/N_2_ baseline, moving from –80.2 MPa to –107.1 MPa. In stark contrast, the SiH_4_/N_2_/NH_3_ system exhibits a fluctuating pattern, with compressive stress dipping from –117.5 MPa to –139.2 MPa before relaxing back to −111.8 MPa. Given the strictly constrained sub-200°C thermal budget, substrate heating triggers negligible thermal relaxation within the rigid dielectric network. The presence of NH_3_—rather than the modest temperature variations—dictates the overall stress profile, consistently imparting a stronger compressive characteristic. This underscores the chemical dominance outlined earlier: the hyper-reactive radicals derived from NH_3_ drive the deposition kinetics. Through a phenomenon analogous to “chemical annealing,” these species efficiently patch microscopic defects and voids during growth, yielding a compacted structural framework that translates macroscopically into amplified compressive stress.

In summary, the incorporation of NH_3_ is found to exert a dual effect on film stress. On the one hand, since NH_3_ is more readily dissociated, a denser film structure is formed, resulting in a tendency toward compressive stress. On the other hand, the introduction of H atoms and the increased porosity lead to a looser film, thereby favoring tensile stress. Therefore, the mechanical regulatory role of NH_3_ is highly dynamic across the pressure spectrum. Depending on the concurrent physical intensity of ion bombardment, NH_3_ is transformed from a “low-pressure densification promoter” (where compressive strain is enhanced) into a “high-pressure porosification catalyst” (where tensile strain is aggravated).

### 3.5. Chemical Bonds

**Figure 10:**
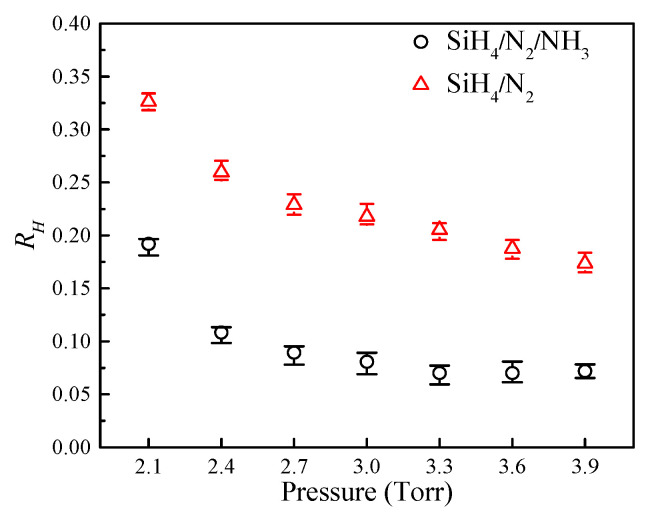
Variation in *R_H_* with pressure for the two systems.

The *R_H_* serves as a crucial microstructural indicator. Figure 10 illustrates its dependence on chamber pressure. Scaling the pressure from 2.1 Torr to 3.9 Torr triggers a monotonic decline in *R_H_* for both systems: the SiH_4_/N_2_ system drops from 0.33 to 0.17, while the SiH_4_/N_2_/NH_3_ system falls from 0.19 to 0.07. The downward trajectory is attributed to a collision-induced preferential cleavage mechanism in the gas phase. As the process pressure is increased, the reactant concentration per unit volume is raised, and the collision probability between particles is correspondingly increased. At elevated pressures, weaker chemical bonds are selectively ruptured by intensified inter-particle collisions [21]. Since the bond energy of Si–H (~318 kJ/mol) is lower than that of N–H (~391 kJ/mol), Si–H bonds are more easily broken under such conditions. Consequently, as the chamber pressure is increased, *R_H_* is gradually decreased. The reduction in *R_H_* is attributed to the introduction of NH_3_. This is thought to be caused by the significantly lower dissociation energy of NH_3_ compared to that of N_2_, by which NH_3_ can participate more actively in the deposition process of silicon nitride thin films. Additionally, since N–H bonds are inherently present in NH_3_, a notable decrease in *R_H_* is observed upon its introduction.

**Figure 11:**
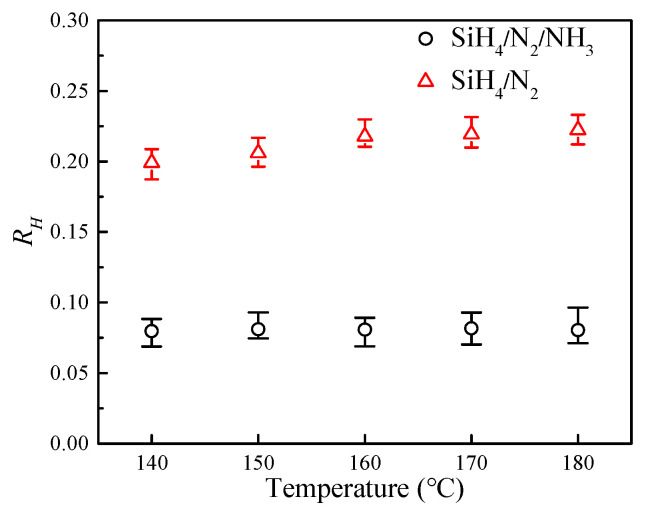
Variation in *R_H_* with temperature for the two systems.

Figure 11 illustrates the variation in the *R_H_* for the two systems at different substrate temperatures. As the temperature is increased from 140 °C to 180 °C, no significant change in *R_H_* is observed for the SiH_4_/N_2_/NH_3_ system. In the SiH_4_/N_2_ system, the *R_H_* value exhibits only a minor fluctuation between 0.20 and 0.22. The *R_H_* of the film shows no clear variation with temperature. This observation further confirms that within this process range, the influence of temperature changes on the formation of the film is limited. The alteration of the bonding configuration between Si–H and N–H in the solid film requires a high thermal activation energy, typically far exceeding 300 °C, to trigger hydrogen desorption [31]. Within the current low-temperature range of 140~180 °C, the available thermal energy is insufficient to reconstruct chemical bonds. In this process window, the chemical bonds of the silicon nitride film are primarily determined by plasma excitation, not thermal activation.

**Figure 12:**
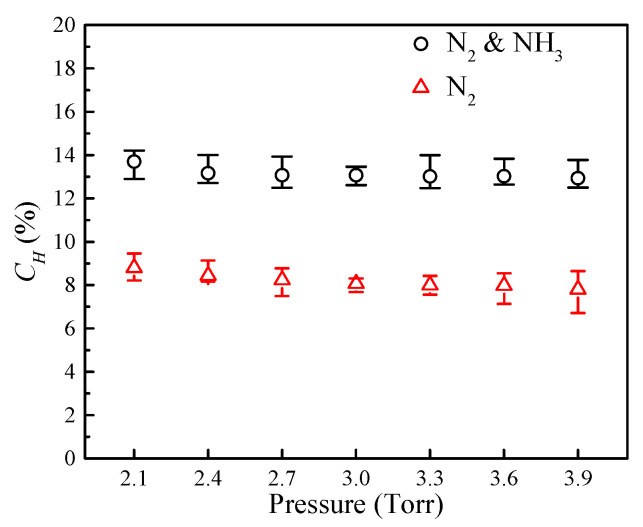
Variation in *C_H_* with pressure for the two systems.

**Figure 13:**
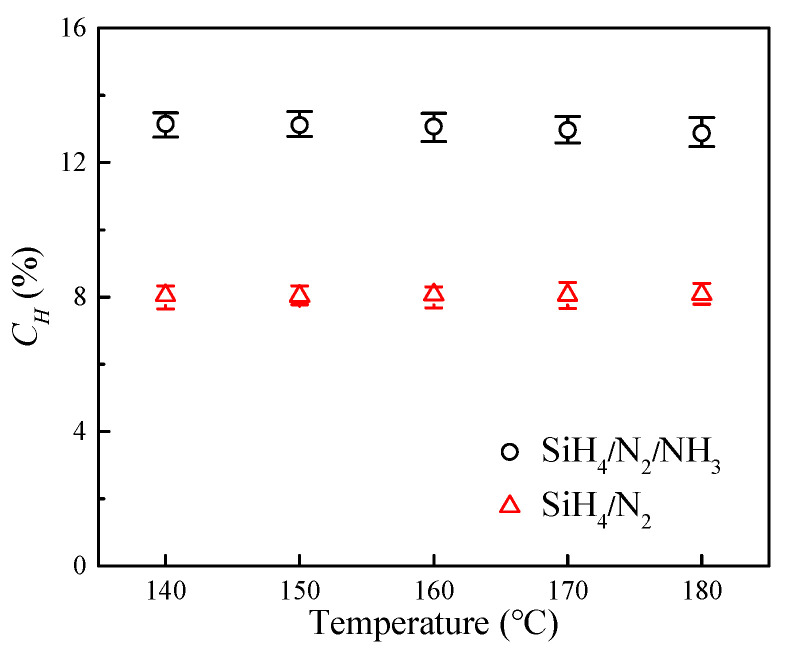
Variation in *C_H_* with temperature for the two systems.

Figure 12 and Figure 13 chronicle the evolution of absolute hydrogen content (*C_H_*) relative to pressure and temperature, respectively. Increasing the pressure from 2.1 to 3.9 Torr systematically suppresses *C_H_* in both systems, reducing it from 8.8% to 7.8% (SiH_4_/N_2_) and from 13.7% to 12.9% (SiH_4_/N_2_/NH_3_). This depletion is intrinsically linked to extended molecular residence times and heightened collision probabilities within high-pressure plasmas. Such conditions encourage premature gas-phase recombination reactions, converting atomic hydrogen into volatile H_2_ molecules that are pumped away. On the contrary, Figure 12 shows that *C_H_* shows no significant change within the substrate temperature range of 140 °C to 180 °C. This reinforces the premise that bulk hydrogen incorporation is an artifact of plasma chemistry—dictated primarily by the initial feed gas composition and RF ionization—rather than thermal desorption. The *C_H_* is drastically elevated by the addition of NH_3_. The reason is that, acting as a highly efficient hydrogen donor, the facile plasma-induced fragmentation of NH_3_ injects massive quantities of H radicals into the deposition environment, inevitably trapping a higher density of hydrogen within the final film.

The introduction of NH_3_ not only significantly enhances the total hydrogen content of the film as an efficient hydrogen source but also reconstructs the internal bonding structure by releasing abundant hydrogen radicals and inducing highly active cross-linking reactions, specifically decreasing the relative ratio of Si–H to N–H bonds. Moreover, the hydrogen increment contributed by NH_3_ exhibits high stability under varying pressure and low-temperature conditions. This indicates that within the current low-temperature process window, both the hydrogen content and bonding state of the film are mainly influenced by the plasma-phase dissociation chemistry, thereby demonstrating corresponding stability against chamber pressure fluctuations and substrate temperature variations.

Compared to temperature, a more sensitive influence on film properties is observed to be exerted by pressure. The core mechanism is attributed to the significant shortening of the mean free path of gas molecules under elevated pressure, which increases collision probabilities in the plasma and consequently reshapes the kinetic balance between gas-phase and surface reactions. First, the reduced mean free path enhances secondary collisions and recombination of reactive radicals in the gas phase. As a result, the effective precursor flux reaching the surface is sharply decreased, and the deposition rate is significantly lowered. Second, although the physical bombardment by ions is weakened under high pressure, the collision frequency of reactive species with the surface is greatly increased. This strengthens chemical dehydrogenation reactions on the surface, promotes hydrogen desorption, and leads to a clear reduction in the hydrogen content of the film.

Compared with the SiH_4_/N_2_ system, the introduction of NH_3_ results in a decrease in the *R_H_* value, indicating that more N is incorporated into the film. This observation is consistent with the increase in deposition rate associated with the addition of NH_3_. Meanwhile, the influence of the *R_H_* value on film performance is primarily reflected in the refractive index. In general, a lower *R_H_* value is accompanied by a lower refractive index. Thus, in the SiH_4_/N_2_ system, the *R_H_* value is reduced as the chamber pressure is increased, and the refractive index is decreased accordingly; conversely, the *R_H_* value is increased as the temperature is raised, followed by an increase in the refractive index. In the SiH_4_/N_2_/NH_3_ system, however, no significant variation in the refractive index is observed. which can be related to the introduction of NH_3_. which modifies the film density, allowing density to become the dominant factor. The introduction of NH_3_ modifies the overall film growth process, thereby leading to a corresponding change in the refractive index.

Furthermore, the non-monotonic change in film uniformity with pressure is explained by the competition between gas diffusion and local reaction rates. In systems without ammonia, uniformity is optimized at moderate pressure due to a balanced mean free path and reactant distribution, while at high pressure, diffusion is suppressed and uniformity is degraded. When ammonia is introduced, the dynamics are altered. At intermediate pressure, a strong “depletion effect” from the center to the edge is caused by local consumption of highly reactive NH_x_ radicals, which increases non-uniformity. However, at even higher pressure, improved uniformity is restored through enhanced collisional quenching and longer gas residence time, which promote a more homogeneous reactant distribution. Notably, the most significant improvement in uniformity is achieved when ammonia is used under low-pressure conditions. This indicates that a long mean free path is essential for ensuring the uniform dispersion of highly reactive fragments across the wafer surface.

Compared with the SiH_4_/N_2_ system, the introduction of NH_3_ also leads to distinct stress behaviors in the film under different chamber pressures. At low chamber pressure, the film tends to exhibit compressive stress, while at high chamber pressure, it shifts toward tensile stress. The influence of NH_3_ on film stress is primarily attributed to two factors. First, NH_3_ dissociates more readily, resulting in a denser silicon nitride film, which promotes compressive stress. Second, the incorporation of NH_3_ can increase *C_H_* in the film, and these hydrogen species tend to induce contraction during cooling, thereby driving the film toward tensile stress. The stress variations observed under different pressure conditions are likely the combined result of these two competing mechanisms.

## 4. Conclusions

In this study, we systematically investigated low-temperature PECVD of silicon nitride films using SiH_4_/N_2_ and SiH_4_/NH_3_/N_2_ reaction systems, aiming to establish a theoretical framework and a process window suitable for mass production. The introduction of NH_3_, which has a lower dissociation energy than N_2_, significantly enhances deposition dynamics. Specifically, adding NH_3_ increased the deposition rate from 18.2 to 39.1 Å/s at 2.1 Torr, while improving thickness uniformity by 1.07 percentage points. The structural and mechanical properties of the films were found to depend strongly on the interplay between NH_3_ addition and chamber pressure. At lower pressures, reactive NH_3_ radicals promote the formation of a highly cross-linked, compact Si–N network, which raises the refractive index and enhances compressive stress. At higher pressures, compared to the pure N_2_ system, NH_3_ leads to an increase in hydrogen bonds, thereby shifting the film stress toward a more tensile direction. Furthermore, film properties depend more significantly on chamber pressure than on substrate temperature, since pressure directly governs plasma collision rates and dynamics, making thermal effects secondary. This mechanism is evidenced by the evolution of Si–H and N–H bonds: increasing the pressure from 2.1 to 3.9 Torr reduces the hydrogen content (*C_H_*) by 0.8%, whereas raising the temperature from 140 °C to 180 °C yields a negligible reduction of only 0.3%.

## Figures and Tables

**Table 1:** Baseline deposition parameters for silicon nitride film.

	SiH_4_ (sccm)	N_2_ (sccm)	NH_3_ (sccm)	Temperature (°C)	Pressure (Torr)	RF Power (W)	Gap (mm)
SiH_4_/N_2_/NH_3_	300	10,000	200	160	3	600	16
SiH_4_/N_2_	300	10,000	0	160	3	600	16

## Data Availability

The original contributions presented in this study are included in the article. Further inquiries can be directed to the corresponding author.

## References

[1] Rathinavelu V., Upadhyay V.V., Prabagaran S., Govindarajan S., Verma A., Soudagar M.E.M., Vinayagam M., Alotaibi M.A., Seikh A.H. (2025). Texturing of silicon nitride passivation layers on functional behaviour study of polycrystalline silicon (p-Si) made with plasma enhanced chemical vapour deposition. J. Mater. Sci.-Mater. Electron..

[2] Zhang Y.H., Li J.Y., Peng H., Yang H., Lu L., Zhang S.D. (2025). Investigation of passivation layers for self-aligned top-gate amorphous InGaZnO thin-film transistors with metal-reacted low-resistance source/drain. Proceedings of the 2025 9th IEEE Electron Devices Technology & Manufacturing Conference, EDTM.

[3] Kempf E., Calvo M., Domengie F., Monfray S., Orobtchouk R. (2020). Low Temperature SiN Waveguides Optimization for Photonic Platform. Proceedings of the International Conference on Transparent Optical Networks (ICTON).

[4] Taka H., Suzuki K., Tsujioka N., Murakami S. (2015). Development of high-quality low-temperature PECVD-SiN films by organosilane. Proceedings of the 2015 International 3D Systems Integration Conference (3DIC).

[5] Ghosh M., Bulkin P., Silva F., Johnson E.V., Florea I., Funes-Hernando D., Tanguy A., Renard C., Vaissiere N., Decobert J. (2022). Ultrathin Ge epilayers on Si produced by low-temperature PECVD acting as virtual substrates for III-V/c-Si tandem solar cells. Sol. Energy Mater. Sol. Cells.

[6] Lai T.L., Lee Y.F., Hsu Y.H., Tsai C.P., Huang C.K., Liu C.Y. (2023). Low-temperature PECVD silicon-nitride passivation for perovskite solar cell. Mater. Chem. Phys..

[7] Martyniuk M., Antoszewski J., Musca C.A., Dell J.M., Faraone L. (2006). Environmental stability and cryogenic thermal cycling of low-temperature plasma-deposited silicon nitride thin films. J. Appl. Phys..

[8] Sahu B.B., Yin Y.Y., Tsutsumi T., Hori M., Han J.G. (2016). The role of plasma chemistry on functional silicon nitride film properties deposited at low-temperature by mixing two frequency powers using PECVD. Phys. Chem. Chem. Phys..

[9] Jehanathan N. (2007). Thermal Stability of Plasma Enhanced Chemical Vapor Deposition Silicon Nitride Thin Films. Master’s Thesis.

[10] Murata T., Miyagawa Y., Isaki R., Shibata T., Matsuda R., Tsujiuchi M., Takeuchi Y., Ueno S., Matsuura M., Asai K. (2009). Effect of NH_3_-Free Silicon Nitride for Protection Layer of Magnetic Tunnel Junction on Magnetic Properties of Magnetoresistive Random Access Memory. Jpn. J. Appl. Phys..

[11] Holmes M.R., Liu S., Keeley J., Jenkins M., Hawkins A.R. (2011). Hollow Waveguides with Low Intrinsic Photoluminescence Fabricated with PECVD Silicon Nitride and Silicon Dioxide Films. IEEE Photonics Technol. Lett..

[12] Meitine M., Sazonov A. (2003). Low Temperature PECVD Silicon Oxide for Devices and Circuits on Flexible Substrates. MRS Proc..

[13] Huang W.D., Wang X.H., Sheng M., Xu L.Q., Stubhan F., Luo L., Feng T., Wang X., Zhang F.M., Zou S.C. (2003). Low temperature PECVD SiNx films applied in OLED packaging. Mater. Sci. Eng. B-Solid State Mater. Adv. Technol..

[14] Oh S.J., Ma B.S., Yang C., Kim T.S. (2022). Intrinsic Mechanical Properties of Free-Standing SiNx Thin Films Depending on PECVD Conditions for Controlling Residual Stress. ACS Appl. Electron. Mater..

[15] Ning J., Tang Z., Chen L., Li B., Wu Q., Sun Y., Zhou D. (2024). Impact of H-related chemical bonds on physical properties of SiNx:H Films deposited via plasma-enhanced chemical vapor deposition. Electronics.

[16] Wang Y.H., Lin J., Huan C.H.A. (2003). Structural and optical properties of a-Si:H/nc-Si:H thin films grown from Ar-H2-SiH4 mixture by plasma-enhanced chemical vapor deposition. Mater. Sci. Eng. B.

[17] Dongling L., Xiaofei F., Zhiyu W., ZhengGuo S., Yin S. (2016). Stress control of silicon nitride films deposited by plasma enhanced chemical vapor deposition. Optoelectron. Lett..

[18] Hsiao S.-N., Nakane K., Tsutsumi T., Ishikawa K., Sekine M., Hori M. (2021). Influences of substrate temperatures on etch rates of PECVD-SiN thin films with a CF4//H2 plasma. Appl. Surf. Sci..

[19] Semenova O., Kozelskaya A., Li Z.-Y., Yu Y.-D. (2015). Mechanical strains in pecvd SiNx:H films for nanophotonic application. Chin. Phys. B.

[20] Lanford W.A., Rand M.J. (1978). The hydrogen content of plasma-deposited silicon nitride. J. Appl. Phys..

[21] Zhou J., Huang J., Liao J., Guo Y., Zhao Z., Liang H. (2021). Multi-field simulation and optimization of SiNx:H thin-film deposition by large-size tubular LF-PECVD. Sol. Energy.

[22] Xu X., He Q., Fan T., Jiang Y., Huang L., Ao T., Ma C. (2013). Hard and relaxed a-SiNxHy films prepared by PECVD: Structure analysis and formation mechanism. Appl. Surf. Sci..

[23] Lee M.-J., Kar J.P., Lee T.I., Lee D., Choi D.-K., Cho J.-H., Myoung J.-M. (2011). Investigation of optical and compositional properties of thin SiNx:H films with an enhanced growth rate by high frequency PECVD method. Vacuum.

[24] El Amrani A., Bekhtari A., Mahmoudi B., Lefgoum A., Menari H. (2011). Experimental study of the effect of process parameters on plasma-enhanced chemical vapour deposition of silicon nitride film. Vacuum.

[25] Mäckel H., Lüdemann R. (2002). Detailed study of the composition of hydrogenated SiNx layers for high-quality silicon surface passivation. J. Appl. Phys..

[26] Alamgeer, Yousuf H., Khokhar M.Q., Jony J.A., Rahman R.U., Hassan S.A.u., Kim Y., Pham D.P., Park S., Yi J. (2024). Improving the optical properties of SiNx:H thin film by optimizing NH3:SiH4 gas ratio using plasma-enhanced chemical vapor deposition. Energy Technol..

[27] Benoit D., Morin P., Regolini J. (2011). Determination of silicon nitride film chemical composition to study hydrogen desorption mechanisms. Thin Solid Film..

[28] Belyansky M., Klymko N., Madan A., Mallikarjunan A., Lai C.W. (2005). Stress Generation in PECVD Silicon Nitride Thin Films for Microelectronics Applications.

[29] Yang Y., Liu Y., Dell J.M. (2009). Effect of heat treatment on internal stresses in PECVD SiNxHy thin films. Proceedings of the 2008 Conference on Optoelectronic and Microelectronic Materials and Devices.

[30] Kim J., Jeong W., Lee S., Jeong S., Park S.H.K. (2021). Effect of H2 addition during PECVD on the moisture barrier property and environmental stability of H:SiNx film. J. Am. Ceram. Soc..

[31] Bakardjieva V., Beshkova G., Vitanov P., Alexieva Z. (2005). Effect of rapid thermal annealving on the properties of µPCVD and PECVD silicon nitride thin films. J. Optoelectron. Adv. Mater..

